# Accuracy and Readability of Chat Generative Pre-Trained Transformer-4 Omni in Answering Ophthalmology Patient Questions

**DOI:** 10.1016/j.xops.2025.101007

**Published:** 2025-11-11

**Authors:** Nikoo Hamzeh, Alcina K. Lidder, Robert S. Feder, Emmanuel A. Sarmiento, Rukhsana G. Mirza, Avrey J. Thau, Angelo P. Tanna

**Affiliations:** Department of Ophthalmology, Northwestern University Feinberg School of Medicine, Chicago, Illinois

**Keywords:** Artificial intelligence, ChatGPT-4o, Ophthalmology, Patient questions, Readability

## Abstract

**Purpose:**

To assess the quality of Chat Generative Pre-Trained Transformer-4 Omni (ChatGPT-4o) responses to questions submitted by patients through Epic MyChart.

**Design:**

Retrospective cross-sectional study.

**Participants:**

One hundred sixty-five patients who submitted ophthalmology-related questions via Epic MyChart.

**Methods:**

Questions asked by ophthalmology clinic patients related to the subspecialties of glaucoma, retina, and cornea via the Epic MyChart at a single institution were evaluated. Nonclinical questions were excluded. Each question was submitted to ChatGPT-4o twice, first without limitations and then after priming the large language model (LLM) to respond at a sixth-grade reading level. The ChatGPT-4o output and subsequent conversations were graded by 2 independent ophthalmologist reviewers as “accurate and complete,” “incomplete,” or “unacceptable” with respect to the quality of the output. A third subspecialist reviewer provided adjudication in cases of disagreement. Readability of the ChatGPT-4o output was assessed using the Flesch–Kincaid Grade Level and other readability indices.

**Main Outcome Measures:**

Quality and readability of answers generated by ChatGPT-4o.

**Results:**

Two hundred eighty-five queries asked by 165 patients were analyzed. Overall, 220 (77%) responses were graded as accurate and complete, 49 (17%) as incomplete, and 16 (6%) as unacceptable. The initial 2 reviewers agreed in 87% of the responses generated by ChatGPT-4o. The overall mean Flesch–Kincaid reading grade level was 12.1 ± 2.1. When asked to respond at a sixth-grade reading level, 242 (85%) responses were graded as accurate and complete, 38 (13%) were incomplete, and 5 (2%) were graded as unacceptable.

**Conclusions:**

Chat Generative Pre-Trained Transformer-4 Omni usually provides accurate and complete answers to the questions posed by patients to their glaucoma, retina, and cornea subspecialists. A substantial proportion of the responses were, however, graded as incomplete or unacceptable. Chat Generative Pre-Trained Transformer-4 Omni responses required a 12th-grade education level as assessed by Flesch–Kincaid and other readability indices, which may make them difficult for many patients to understand; however, when prompted to do so, the LLM can generate responses at a sixth-grade reading level without a compromise in response quality. Chat Generative Pre-Trained Transformer-4 Omni can potentially be used to answer clinical ophthalmology questions posed by patients; however, additional refinement will be required prior to implementation of such an approach.

**Financial Disclosure(s):**

Proprietary or commercial disclosure may be found in the Footnotes and Disclosures at the end of this article.

When patients have clinical questions they wish to have answered by their ophthalmologist, they often pose the questions using web-based communication applications such as Epic MyChart that is a component of the electronic medical record (EMR) system. Advances in artificial intelligence, neural networks, and deep learning algorithms have begun to influence medical care. Chat Generative Pre-Trained Transformer-4 Omni (ChatGPT-4o) (OpenAI) is a generative artificial intelligence language chatbot capable of answering questions on a wide range of topics, including ophthalmology.^1^

Previous studies indicated that the performance of ChatGPT varies and requires additional research and improvement, particularly in medicine and ophthalmology-related subjects.^2^, ^3^, ^4^

Chat Generative Pre-Trained Transformer-4 Omni was released on May 13, 2024, with enhanced capabilities over earlier iterations. It is notably better at interpreting visual inputs and responding to audio inputs, making interactions feel more like conversations with a human.^5^

Chat Generative Pre-Trained Transformer has been shown to accurately answer a large proportion of Ophthalmic Knowledge Assessment Program questions and ophthalmology questions from StatPearls, an education platform that offers a large bank of peer-reviewed questions designed for use by medical professionals.^6^ We hypothesized that ChatGPT-4o can produce complete and accurate answers in response to patients’ questions posed to their ophthalmologists via Epic MyChart.

## Methods

This study adhered to the tenets of the Declaration of Helsinki and was conducted in compliance with the Health Insurance Portability and Accountability Act. The study was approved by the Northwestern University Institutional Review Board, and the requirement for written informed consent was waived due to its retrospective nature and the use of de-identified data.

A selection of questions asked by patients who received care from 1 glaucoma specialist, 2 retina specialists (1 medical and 1 surgical), and 1 cornea specialist at a tertiary care ophthalmology center via the Epic MyChart platform in Epic between January 2023 and January 2024, were retrospectively evaluated. Patients were identified through a review of the clinic schedule between August 2023 and December 2023. Each patient’s chart was reviewed to identify messages sent within the specified timeframe (January 2023 to January 2024) under the “Encounters” section in Epic. Clinical questions were copied and submitted to ChatGPT-4o. This process was repeated until the clinical questions became repetitive. Many patients asked similar questions about common topics such as dry eye and floaters. We used a convenience sample and stopped reviewing questions once substantial repetition of the same question type was observed. Nonclinical questions were excluded. If a question required prior knowledge regarding the patient’s condition that was available in the EMR and would therefore have been known by staff or attending physicians responding to the same question, a brief explanation was added to the question posed to ChatGPT-4o in order to provide necessary context. For example, in the case of a patient undergoing pterygium surgery who asked about stopping aspirin, the following was entered: “Patient’s Question (This patient will be having pterygium surgery): My cardiologist wants to know how long I need to be off of the aspirin prior to surgery?”

Each question was submitted to ChatGPT-4o verbatim, as asked by the patient, and the responses were recorded. The responses were graded by 2 independent ophthalmologist reviewers. One general ophthalmologist (N.H.) graded all responses; depending on the subspecialist to whom the question was directed, the second grader was a glaucoma (A.K.L.) or cornea (A.J.T.) subspecialist attending or a vitreoretinal surgery fellow (E.A.S.). Responses were graded using the following classification:

Accurate and complete: the provided answer is medically and scientifically correct, answers the question thoroughly, and can be given to the patient in a clinical setting.

Incomplete: the provided answer is somewhat correct but is not completely thorough and requires further clarification.

Unacceptable: the provided answer is partly or overall, medically, or scientifically incorrect or gives inaccurate information.

A third reviewer with subspecialty expertise in the relevant field (R.S.F., R.G.M., A.P.T.) adjudicated cases of disagreement between the first 2 reviewers. Levels of interrater agreement between the first 2 sets of reviewers were assessed with descriptive statistics and linearly weighted Cohen kappa statistics.

To assess whether requesting responses at a sixth-grade reading level would lead to accurate responses, we asked the same questions again and instructed ChatGPT-4o to provide answers at a sixth-grade reading level, as recommended by the American Medical Association for health-related materials. Two-proportion z-tests were performed to compare the proportions of “accurate and complete,” “incomplete,” and “unacceptable” responses between the standard and sixth-grade reading level responses within each subspecialty. In addition, a chi-square test of independence was performed to evaluate whether the distribution of response gradings (“accurate and complete,” “incomplete,” and “unacceptable”) was different between the standard and sixth-grade reading level responses within each subspecialty.

Readability of the ChatGPT-4o responses were assessed using Flesch–Kincaid Grade Level, Flesch Reading Ease Score, Gunning Fog Index, Coleman–Liau Index, and the Simple Measure of Gobbledygook Index. We assessed the readability scores by inputting the questions into an online readability analysis application (https://readabilityformulas.com). The Felsch–Kincaid Grade Level readability test, for example, uses sentence complexity, choice of words, and textual cohesion to measure the education grade level at which the text can be easily understood.

## Results

**Figure 1:**
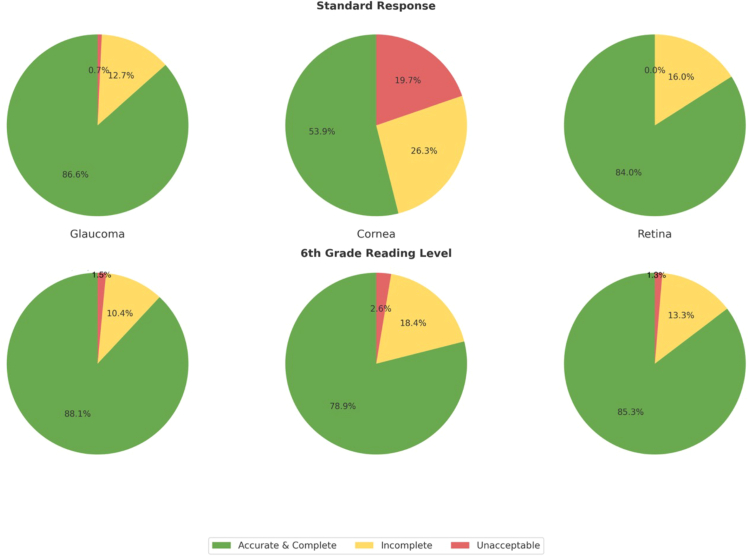
Distribution of ChatGPT-4o response gradings across subspecialties. ChatGPT-4o = Chat Generative Pre-Trained Transformer-4 Omni.

Eight-hundred sixty-seven questions asked by patients were reviewed. Five hundred eighty-two questions were excluded because they were not clinical questions or they were duplicative of previously entered questions. A total of 285 queries asked by 165 patients were posed to ChatGPT-4o. A brief explanation of context was added to 71 questions due to their requirement of prior knowledge of patients’ medical information (e.g., an existing diagnosis or the type of surgery performed recently). The questions were classified into 5 groups. Sixty-one (21.4%) addressed medications and their side effects, 99 (34.7%) focused on procedures and recovery, 74 (26.0%) related to symptoms, 36 (12.6%) to diagnosis, monitoring and prognosis, and 15 (5.3%) were classified as other. The initial 2 reviewers agreed in 87% (249) of the responses reported by ChatGPT-4o. The final reviewers’ gradings, including those adjudicated by the third reviewer, of the ChatGPT-4o output are summarized in the Table 1 and Figure 1. The readability of ChatGPT-4o responses, assessed using various indices, is summarized in Table 2.

**Table 1:** Grading of the ChatGPT-4o Output

Subspecialty	Number of Questions	ChatGPT-4o Output Grading			Linearly Weighted Cohen’s Kappa∗
		Accurate and Complete	Incomplete	Unacceptable	
Standard response					
Overall	285	220 (77.2%)	49 (17.2%)	16 (5.6%)	–
Cornea	76	41 (53.9%)	20 (26.3%)	15 (19.7%)	0.80
Glaucoma	134	116 (86.6%)	17 (12.7%)	1 (0.7%)	0.58
Retina	75	63 (84.0%)	12 (16.0%)	0 (0.0%)	0.43
Sixth-grade reading level					
Overall	285	242 (84.9%)	38 (13.3%)	5 (1.8%)	–
Cornea	76	60 (78.9%)	14 (18.4%)	2 (2.6%)	0.70
Glaucoma	134	118 (88.1%)	14 (10.4%)	2 (1.5%)	0.47
Retina	75	64 (85.3%)	10 (13.3%)	1 (1.3%)	0.54

ChatGPT-4o = Chat Generative Pre-Trained Transformer-4 Omni.

∗ Agreement between initial reviewers.

**Table 2:** Readability Scores of Patient Questions across Subspecialties

Subspecialty	Flesch Reading Ease Score	Gunning Fog Index	Flesch–Kincaid Grade Level	Coleman–Liau Index	Simple Measure of Gobbledygook (SMOG) Index
Glaucoma	37.2 ± 12.7 37, (5–79)	15.4 ± 2.4 15.1, (7–21.9)	11.9 ± 2.0 11.9, (5.1–16.8)	14.6 ± 2.4 14.8, (6.9–20.8)	10.9 ± 1.5 10.6, (5–15.8)
Cornea	48.5 ± 12.3 48, (13–76)	15.2 ± 2.6 15.4, (9–20.6)	11.9 ± 2.3 12.0, (6.0–17.6)	11.8 ± 2 11.9, (6.9–17.3)	10.9 ± 2 11.1, (4.4–14.7)
Retina	40.7 ± 10 41, (4–59)	16.1 ± 2.4 16.1, (10.8–25)	12.5 ± 1.9 12.2, (8.6–18.5)	13.1 ± 1.7 13.1, (9.6–18.6)	11.7 ± 1.6 11.8, (8–17.2)
Overall	41.1 ± 12.8 41, (4–79)	15.5 ± 2.5 15.6, (7–25)	12.1 ± 2.1 12.0, (5.1–18.5)	13.5 ± 2.4 13.4, (6.9-20.8)	11.1 ± 1.7 11.1, (4.4–17.2)

Data are presented as mean ± standard deviation, median, range.

Two-proportion z-tests showed a statistically significant improvement in the quality of ChatGPT-4o output in the cornea category, with an increase in accurate and complete responses (*P* = 0.001) and a significant decrease in unacceptable responses (*P* = 0.0008) when ChatGPT-4o was primed to respond at a sixth-grade reading level. No statistically significant differences were observed in the glaucoma or retina subspecialties across any response categories (*P* > 0.05 in all categories). The chi-square test showed a significant difference in the distribution of responses for the cornea subset (*P* = 0.0007). There was no significant difference between the distribution of responses in the glaucoma and retina categories (*P* = 0.73 and *P* = 0.55, respectively).

### Glaucoma

A total of 134 questions directed to a glaucoma subspecialist were analyzed. Among these, 110 responses (82%) were graded as “accurate and complete” by both initial reviewers, while 11 (8%) were graded as “incomplete.” There was disagreement between the reviewers in 13 responses (10%). After adjudication by the third reviewer, 6 responses were graded as “accurate and complete,” 6 as “incomplete,” and 1 as “unacceptable.” The reviewers agreed on 90% of the ChatGPT-4o responses. The weighted Cohen kappa was 0.58, indicating a moderate level of agreement. The readability analysis showed that the mean Flesch–Kincaid Grade Level was 11.9 ± 2.0 (range 5.1–16.8).

We posed the questions to ChatGPT-4o again and instructed the model to respond at a sixth-grade reading level. In this setting, 107 responses (80%) were rated as “accurate and complete” by both initial reviewers, while 7 responses (5%) were graded as “incomplete.” One question (1%) was rated as “unacceptable” by both reviewers. Disagreements occurred in 19 responses (14%). After adjudication by the third reviewer, 11 responses were graded as “accurate and complete,” 7 as “incomplete,” and 1 as “unacceptable.” Overall, reviewers agreed on 86% of the ChatGPT-4o responses, with a weighted Cohen kappa of 0.47, indicating a moderate level of agreement.

### Cornea

A total of 76 questions directed to a cornea subspecialist were analyzed. Among these, 37 responses (49%) were graded as “accurate and complete” by both reviewers, while 12 (16%) were graded as “incomplete” and 15 (20%) as “unacceptable.” There was disagreement between the initial reviewers in 12 responses (16%). After adjudication by the third reviewer, 4 responses were graded as “accurate and complete,” and 8 as “incomplete.” The reviewers agreed on 84% of the ChatGPT-4o responses. The weighted Cohen kappa was 0.80, indicating excellent agreement. The readability analysis showed that the mean Flesch–Kincaid Grade Level was 11.9 ± 2.3 (range 6.0–17.6).

When prompting ChatGPT-4o to generate answers at a sixth-grade reading level, 57 responses (75%) were graded as “accurate and complete” by both reviewers, while 7 (9%) were graded as “incomplete” and 2 (3%) as “unacceptable.” There was disagreement between the initial reviewers in 10 responses (13%). After adjudication by the third reviewer, 3 responses were graded as “accurate and complete,” and 7 as “incomplete.” The reviewers agreed on 87% of the ChatGPT-4o responses. The weighted Cohen kappa was 0.70, indicating excellent agreement.

### Retina

A total of 75 questions directed to a retina subspecialist were analyzed. Among these, 58 responses (77%) were graded as “accurate and complete” by both reviewers, while 6 (8%) were graded as “incomplete.” There was disagreement between the reviewers regarding 11 responses (15%). After adjudication by the subspecialist expert reviewer, 5 responses were graded as “complete” and 6 as “incomplete.” The reviewers agreed on 85% of the ChatGPT-4o responses. The weighted Cohen kappa was 0.43, indicating a moderate level of agreement. The readability analysis showed that the mean Flesch–Kincaid grade level was 12.5 ± 1.9 (range 8.6–18.5).

After prompting ChatGPT-4o to provide answers at a sixth-grade reading level, 61 responses (81%) were graded as “accurate and complete” by both reviewers, while 3 (4%) were graded as “incomplete.” One response (1%) was graded as “unacceptable” by both initial reviewers. There was disagreement between the reviewers regarding 10 responses (13%). After adjudication by the subspecialist expert reviewer, 3 responses were graded as “accurate and complete” and 7 as “incomplete.” The reviewers agreed on 87% of the ChatGPT-4o responses. The weighted Cohen kappa was 0.54, indicating a moderate level of agreement.

## Discussion

We found that ChatGPT-4o provided accurate and complete answers to most clinical questions posed by glaucoma, retina, and cornea service patients via the MyChart Epic platform. Chat Generative Pre-Trained Transformer-4 Omni responses were presented at a 12th-grade level, which requires a high school education to comprehend. When promoted to generate responses at a sixth-grade level, we did not observe a significant difference in the proportion of responses that were graded as accurate and complete in the retina and glaucoma categories; however, there was significant improvement in the cornea category. This was an interesting and unexpected finding. It appears that when the large language model (LLM) generates answers in the cornea category, fewer inaccuracies may be introduced when the complexity of the response is capped at a sixth-grade level.

With their increasing popularity, the potential utility of the use of ChatGPT and other LLMs in answering patient questions is an important area of investigation. Notably, ChatGPT-3.5 achieved nearly passing scores in all 3 steps of the United States Medical Licensing Examination without any input from trainers.^7^ Chat Generative Pre-Trained Transformer 3.5, ChatGPT-4o, and Bing Chat performed similarly to the mean performance of human test takers, based on historical data of the proportion of subscribers who correctly answered questions from the American Academy of Ophthalmology’s Basic and Clinical Science Course Self-Assessment Program (personal communication, Louis Z Cai, MD, February 25, 2025).^4^

Biswas et al reviewed 70 original reports, all including use of artificial intelligence–based LLMs in ophthalmology. In their analysis of previously published reports, ChatGPT-4 responded correctly to an average of 75.9% of text-based ophthalmology examination questions and performed significantly better than earlier iterations of the same LLM.^1^^,^^8^^,^^9^ In a comparison among different LLMs and human experts, human experts performed best in diagnosing ophthalmic diseases, while ChatGPT-4 and Bing Chat were most accurate in providing information and answering questions.^1^ In our study, 77% of the ChatGPT-4o responses to patient questions were graded as accurate and complete.

Large language models have also been assessed for their accuracy in answering questions asked on online forums, simulated patient complaints and triage, “frequently asked questions” obtained from various online sources of information such as hospital and professional society websites, or questions asked by Google users.^10^, ^11^, ^12^, ^13^, ^14^, ^15^, ^16^, ^17^, ^18^, ^19^, ^20^, ^21^, ^22^, ^23^, ^24^, ^25^ Biswas et al^1^ observed that among previous reports on the accuracy of LLMs in answering simulated patient questions and providing information, ChatGPT-4 responses were judged to have been accurate in a mean of 84.6% of queries. To the best of our knowledge, only one previous study evaluated real patient questions.^26^ Al Sharif et al assessed ChatGPT-3.5 and Bard responses to real patient questions sent to oculoplastics specialists via an EMR system. In their study, ChatGPT-3.5 substantially outperformed Bard in most categories, providing "comprehensive" answers to 71.4% of the questions patients asked, while its responses were partially or completely incorrect in <10% of cases.^26^

In this study, ChatGPT-4o produced “accurate and complete” answers in response to 77% of questions overall; however, only 48% of questions asked by patients regarding corneal and anterior segment diseases were judged to have been “accurate and complete.” Moshirfar et al investigated the performance of ChatGPT-3.5 and 4 and compared it to the historical performance of human users in answering ophthalmology StatPearls questions. Chat Generative Pre-Trained Transformer 4 performed better than previous human respondents in the anterior segment category overall, while humans performed better in the lens and cataract-related subcategory questions.^6^ In our study, 43% of the questions in the cornea category were related to cataract surgery, including different intraocular lens types. The lower success rate of ChatGPT-4o in answering cornea related questions in our study may be driven by a lack of access to the most up-to-date material in the field or the complex nature of such questions which may require more advanced synthesis of knowledge.

In this study, ChatGPT-4o output was at about the 12th-grade reading level in all categories as measured by Flesch–Kincaid Grade Level, Flesch Reading Ease, and Gunning Fog Index, and other indices, suggesting the requirement for at least a high school education for the patients to fully understand most answers. Similarly, most studies indicate that the online patient education materials available on ophthalmology topics including glaucoma and retina are above the recommended sixth-grade reading level, with substantial variation in both content and quality.^2^^,^^27^, ^28^, ^29^, ^30^, ^31^ Accordingly, we prompted ChatGPT-4o to provide responses generated at a sixth-grade reading level. This aligns with findings from a recent study, which showed that prompt engineering—such as including explicit instructions to write at a sixth-grade level using readability formulas can significantly improve the readability of LLM-generated materials.^32^ Interestingly, these simplified ChatGPT-4o responses did not significantly differ in quality as graded by reviewers in the glaucoma and retina subspecialties and, in fact, improved in the cornea subspecialty.

The current study has several limitations. First, we extracted real questions patients had asked their ophthalmologists who were already familiar with their ophthalmic conditions and medical history. Although ChatGPT-4o was primed with a brief explanation for about a quarter of the questions, the answers may have been more complete if the patients’ complete EMR data have been available. Second, we used subjective grading performed by 2 ophthalmologists in each category to assess ChatGPT-4o responses, with moderate to excellent intergrader agreement. Third, ChatGPT-4o has access to a limited database. Although ChatGPT-4o has the ability to perform web-based searches, its answers might have been affected by lack of access to the most up-to-date body of knowledge. The study was conducted at a single tertiary care center and involved only one physician in each of the 3 subspecialties studied. Therefore, the results may not be generalizable to other settings. Some types of questions were asked by multiple patients. These were submitted to ChatGPT-4o more than once; however, after multiple duplicates of the same question type were detected, subsequent questions judged to have been very similar were excluded. This was done in a subjective fashion; therefore, the questions studied may be more varied and not fully representative of those observed in typical clinical settings. The grading system used to categorize responses as complete, incomplete, or unacceptable was subjective and did not utilize a validated instrument. Another limitation of this study is that we only used ChatGPT-4o to evaluate responses and did not test other widely available LLMs. We selected ChatGPT-4o because previous iterations of this LLM were found to provide the most accurate responses to ophthalmology questions.

In conclusion, ChatGPT-4o usually provides accurate and complete answers to questions posed by patients to their glaucoma, retina, and cornea subspecialists. A substantial proportion of the responses were; however, graded as incomplete or unacceptable, creating a barrier to implementation of such technology in clinical practice. Chat Generative Pre-Trained Transformer-4 Omni responses required a 12th-grade reading level as assessed by Flesch–Kincaid and other readability indices, which may make them difficult for many patients to understand. Prompting the LLM to respond at a sixth-grade reading level did not result in a loss of response quality. Chat Generative Pre-Trained Transformer-4 Omni can potentially be used to answer ophthalmology clinical questions posed by patients; however, additional refinement and further research will be required.
