# Supplementary material for: Accuracy and Readability of Chat Generative Pre-Trained Transformer-4 Omni in Answering Ophthalmology Patient Questions
Source: Ophthalmol Sci. 2025 Nov 11;6(2):101007. doi: 10.1016/j.xops.2025.101007 (PMC12756631; doi:10.1016/j.xops.2025.101007)
Supplement: Supplementary Material [file mmc1.pdf]

Supplemental Appendix: Sample Patient Questions and ChatGPT-4o Generated Responses

COMPLETE ANSWERS:

Patient's Question: "I applied one drop of Rhopressa in my right eye for the first time last night after picking it up yesterday. I woke up this morning with blurry vision, redness, and light tearing. I have not noticed any pain. Blurry vision - slight improvement - 10%. Redness. Light dryness. No tearing. No pain."

ChatGPT-4o Answer: It sounds like you are experiencing some common side effects of Rhopressa (netarsudil), which is a medication used to lower intraocular pressure in glaucoma. Here's how to address your symptoms:

1. **Blurry Vision:**
    - **Common Side Effect:** Blurry vision can occur with the use of Rhopressa, especially when starting the medication. It is usually temporary and should improve as your eyes adjust to the medication.
    - **Monitoring:** Since you have noticed a slight improvement, continue to monitor your vision. If the blurriness persists or worsens, contact your ophthalmologist.
2. **Redness:**
    - **Expected Reaction:** Redness is a common side effect of Rhopressa and can occur in many patients. It typically decreases with continued use.
    - **Management:** If the redness is mild and not accompanied by pain or significant discomfort, it is generally safe to continue the medication. Using lubricating eye drops (preservative-free artificial tears) can help soothe your eyes.
3. **Light Dryness:**
    - **Dry Eye Management:** Light dryness can be managed with the use of artificial tears. Apply these drops as needed throughout the day to keep your eyes comfortable.
4. **No Tearing or Pain:**
    - **Positive Signs:** The absence of tearing and pain is a good sign and indicates that you are not experiencing a severe reaction to the medication.

Next Steps:

- **Continue Monitoring:** Keep using the medication as prescribed and monitor your symptoms. Side effects often diminish over time.
- **Symptom Relief:** Use preservative-free artificial tears to manage dryness and redness. Apply them as needed for comfort.
- **Contact Your Doctor:** If the blurry vision, redness, or dryness persist beyond a few days, or if you develop new symptoms such as significant pain, increased tearing, or worsening vision, contact your ophthalmologist for further evaluation.

If you have any further questions or concerns, please let me know. Your comfort and eye health are important, and we want to ensure you receive the best care possible.

Patient's Question (This patient has a non-healing corneal ulcer): "My eye is red, swollen, sensitive to light, crusty, watering, and somewhat painful. Is this to be expected with the Prokera Slim or is something awry? "

ChatGPT-4o Answer: With the symptoms you're describing—redness, swelling, light sensitivity, crusting, tearing, and pain—it's possible that there's irritation or even an infection that needs attention, especially given your history of a non-healing corneal ulcer.

While **mild discomfort** and some **tearing** can occur with the Prokera Slim, the level of pain, redness, and sensitivity you're experiencing may indicate that the eye needs further evaluation to rule out any complications, such as infection or improper placement.

Here are some steps we'll take to address this:

1. **Examining the Eye**: Let's take a closer look at your eye to check for signs of infection or other issues related to the Prokera placement. We'll examine whether the Prokera Slim is positioned properly and ensure there are no complications from the ulcer.
2. **Possible Adjustments**: If the Prokera Slim is contributing to discomfort or there are signs of an adverse reaction, we may consider repositioning or temporarily removing it.
3. **Symptom Management**: In the meantime, you might benefit from preservative-free lubricating drops to help keep your eye comfortable and reduce dryness around the Prokera.
4. **Medications**: Depending on what we find, we may adjust your medication or prescribe something additional to help manage the symptoms.

Please come in as soon as possible so we can address these symptoms and ensure everything is progressing as expected.

Patient's Question: "These ketorolac drops cause my left eye to sting uncomfortably, though the pain does subside in a few seconds. The pain concerns me. Is this normal and should I continue the medication?"

ChatGPT-4o Answer: It is not uncommon for ketorolac drops to cause a stinging sensation upon application, which typically subsides quickly. However, if the discomfort is severe or persists, it is important to consult your ophthalmologist. They may advise you to continue

the medication if the stinging is mild and transient, or they might adjust your treatment plan if necessary. Do not discontinue the medication without first consulting your doctor.

INCOMPLETE ANSWERS:

Patient's Question (This patient is underwent Cyclophotocoagulation): "Is it okay for me to go back to doing water exercises? They are not strenuous and I can wear glasses while in the pool?"

ChatGPT-4o Answer: After undergoing a Cyclophotocoagulation (CPC) procedure, it's important to follow post-procedure care instructions carefully to ensure proper healing and avoid complications. Here's some guidance regarding returning to water exercises:

General Guidelines Post-CPC:
Avoid Water Exposure: Typically, it's recommended to avoid getting water in the eyes for at least a few weeks after the procedure to reduce the risk of infection.
Healing Time: The healing period can vary, and it's crucial to ensure the eye has adequately healed before engaging in activities that could expose it to water.
Water Exercises:
Low-Intensity Exercises: If the exercises are low-intensity and you can ensure your head remains above water, it may be safer. However, there's still a risk of splashing or accidental exposure.
Protective Eyewear: Wearing glasses can help protect your eyes, but they may not provide complete protection against water exposure.
Consult Your Ophthalmologist:
Personalized Advice: It's best to consult your ophthalmologist before resuming any water-based activities. They can provide personalized advice based on your specific healing progress and eye condition.

Next Steps:

> **Contact Your Ophthalmologist:** Reach out to your ophthalmologist to discuss your desire to resume water exercises and get their approval.
> **Follow-Up Appointment:** Schedule a follow-up appointment if necessary to assess the healing progress of your eye post-CPC.

Final Recommendation:

> **Seek Approval First:** Ensure you get approval from your ophthalmologist before resuming water exercises to avoid any potential complications and ensure proper healing.

If you have any further questions or need additional assistance, please let me know. Your eye health and proper recovery after the CPC procedure are our top priorities.

Patient's Question: " I woke up with a purple eyelid yesterday. Is this a symptom of concern? I was sleeping and didn't bump into anything. Thank you. - 1.eyelid feels slightly heavy 2. Vision is not impaired 3. Have not used anything to heal it. 4. Purple has fallen down to the corner of my eye. 5. Can't tell if it's swollen. Both eyelids fall down overlapping my lower lids. I'm driving to urgent care close by- now. I'd appreciate a dr looking at it. Thank you for your quick response!

ChatGPT-4o Answer: Thank you for the detailed information. A sudden purple discoloration on the eyelid, especially if it appears without an injury, can sometimes indicate minor bleeding under the skin (a form of ecchymosis) or even an allergic reaction or irritation.

Given the **slight heaviness** you're feeling, it's wise to have it checked promptly, especially since the color seems to be shifting. Here's what urgent care or our office will assess:

1. **Check for Underlying Bleeding or Bruising**: Sometimes, small blood vessels can break spontaneously, causing a bruise-like appearance.
2. **Swelling and Inflammation**: They'll examine for signs of swelling, as this could suggest an inflammatory or allergic reaction.
3. **Other Eye Symptoms**: While your vision is unaffected, they'll also check for any other eye-related symptoms to ensure nothing deeper is involved.

If urgent care has any additional findings, feel free to update us, and we'll coordinate any necessary follow-up. Hopefully, it's something minor, but it's good to be cautious.

UNACCEPTABLE ANSWERS:

Patient's Question: "Since the surgery, I have been using PrednisoLONE 4 times a day for 3 weeks, then on Aug 4, in order to correct extreme dryness in my right eye, Dr. [redacted] suggested to try PrednisoLONE 2 times, GenTeal Gel 4 times, and Systane 4 times a day. On the third day of this new regimen and 3.5 weeks after the surgery, my vision in the right eye has not improved, i.e. still very blurry. When I use PrednisoLONE and GenTeal Gel now they hurt my eye so much that my eye tears and I cannot open my eye for almost 20 minutes. So I stopped using them tonight and I am using only Systane 4 times a day, which is very soothing. I hope this is ok."

ChatGPT-4o Answer: Given the discomfort and tearing you're experiencing with PrednisoLONE and GenTeal Gel, it's understandable that you decided to pause those drops. Here are a few considerations to keep your eye comfortable and safe while we troubleshoot this issue:

1. **Temporary Use of Systane**: Continuing with just Systane four times a day for now is fine, especially as it's providing relief without the irritation. Systane is typically soothing and may help alleviate dryness in the short term.
2. **Check for Sensitivity**: It's possible that the PrednisoLONE or GenTeal Gel is causing sensitivity or irritation. Letting us know about this reaction is essential, as we may consider alternative anti-inflammatory or lubricating options.
3. **Follow-Up Evaluation**: Since the vision is still blurry and this new regimen hasn't yet improved dryness or comfort, a follow-up appointment would be beneficial. This will help us reassess the dryness level, check for any inflammation, and adjust your treatment as needed.

Feel free to keep using Systane as needed for comfort until your next visit, and we'll tailor the regimen to ensure it's both effective and comfortable for you. Let us know if there's any worsening in vision or new symptoms in the meantime.

Patient's Question: "On July 15 you performed cataract surgery on my right eye. I had no problems after, but recently I have floater in my right eye. I made an appointment to see you, but the only available time was in May. I don't know if this condition is high priority or I can wait until my appointment. I'd appreciate your prompt response.Thank you"

ChatGPT-4o Answer: Floaters after cataract surgery are fairly common and often harmless, but it's wise to monitor any new changes in your vision. Here's what to consider:

1. **Routine Check-Up**: If the floater is stable, not accompanied by flashes of light, and doesn't interfere significantly with your vision, it's generally safe to wait for your scheduled appointment.
2. **Signs to Watch For**: If you notice any sudden increase in floaters, flashes of light, or a shadow in your vision, it's essential to be seen sooner. These symptoms could indicate a retinal issue that requires prompt attention.

If there are any changes in your symptoms or if you'd like reassurance sooner, let us know, and we'll try to arrange an earlier appointment. Otherwise, monitoring your vision until May should be fine as long as there are no new developments.
